# A Novel *Cortex Phellodendri Chinensis*–Based Carbon Dots Platform for Remarkable Analgesia for Clinical Pain Management

**DOI:** 10.1002/vms3.70090

**Published:** 2024-11-04

**Authors:** Huimin Peng, Xingxing Zeng, Songbai Li, Xin Wang

**Affiliations:** ^1^ Department of Pain Xiangyang Central Hospital Affiliated Hospital of Hubei University of Arts and Science Xiangyan Hubei China

**Keywords:** analgesic, CDs, cytotoxicity, nociception, pain

## Abstract

In this study, we explored the eco‐friendly synthesis of photoluminescent CCDs employing a direct one‐step pyrolysis process, utilizing natural *Cortex Phellodendri Chinensis* as the precursor material and studied their analgesic effect in mice. The synthesized carbon dots underwent comprehensive characterization through a range of spectroscopic and microscopic techniques. These included UV‐Vis, FTIR, fluorescence spectroscopy and HR‐TEM, DLS instruments. HR‐TEM results exhibited the presence of homogenous spherical‐shaped C‐dots of about 3.3 nm without aggregates. Furthermore, the prepared CCDs were studied for their in vivo analgesic effect in mice by performing tail‐immersion, hot plate and acetic acid writhing tests. Also, an MTT assay was performed to assess the in vitro cytotoxicity of CCDs against L929 cells. In vitro cytotoxicity studies revealed that L929 cells exhibited higher cell viability when treated with prepared CCDs. The cellular uptake studies revealed the phase contrast images of MG‐63 cells at wavelength 488 nm clearly depicted the aggregation of green, fluorescent CCDs within the cells while leaving nuclei unobscured. In addition, to the best of our understanding, the results presented in this paper showed that CCDs exhibited an important analgesic effect and enhanced anti‐nociceptive activity, which may be due to stimulation of the opioidergic system. Consequently, CCDs appear to be a viable analgesic alternative for traditional analgesic candidates in pain management.

## Graphical abstract

A one‐step direct pyrolysis method for the production of green photoluminescent, low‐toxic CCDs using natural *Cortex Phellodendron Chinensis*. CCDs exhibit important analgesic effects, which may act as a viable analgesic alternatives for traditional analgesic candidates in pain management.

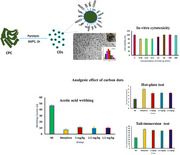

## 1 Introduction

*Chinese Cortex Phellodendri*, commonly known as Huang Bai in traditional Chinese medicine, is the most extensively utilised substance in this ancient healthcare system. It is used in herbal medicines to warfare toxicity, eliminate damp heat, diarrhoea, alleviate consumptive fever, extinguish fire and treat dysentery and other conditions (Chan 1994). It is obtained from the *Phellodendron amurense* Rupr. (*Cortex Phellodendron Amurensis*) or *Phellodendron chinense* Schneid (*Cortex Phellodendron Chinensis*) dried bark. The two plants are substitutable in therapeutic interventions due to their similar chemical compositions. Recent research has shown that the presence of berberine, the primary active metabolite of medicinal herbs, was markedly larger in *P. amurense* Rupr. than in *P. chinense* Schneid (Chan et al. 2007; Chen et al. 2010).

Traditional remedies have made extensive use of *Cortex Phellodendri* as an anti‐inflammatory agent (Park et al. 2007). Recent research demonstrates that *Cortex Phellodendron Amurensis* had in vitro (Park et al. 2007) and in vivo (Mao et al. 2010) anti‐inflammatory properties. Early literature has shown that *P. amurense* Rupr. and *P. chinense* Schneid exhibited distinct anti‐diarrhoeal and anti‐microbial properties; however, their combination resulted in enhanced anti‐inflammatory activity in vivo (Park et al. 2007; Mao et al. 2010).

Carbon dots (CDs) are a family of fluorescent nanoparticles with an average diameter of not less than 10 nm, which can demonstrate quantum dot‐like properties (Liu et al. 2020). CDs have been viewed as prospective replacements for conventional fluorescent dyes because of their broad range of optical features (Xu et al. 2004) and photobleaching resistance (Li et al. 2019). CDs also show lower toxic effects (chemical and cytotoxicity) than most other quantum dots (Yang et al. 2009), indicating their promise for biological applications.

Initially, the synthesis of CDs utilized the top–down methods in which graphite was transformed into graphite oxide through multiple stages involving toxic chemicals (Hummers and Offeman 1958) before even being decomposed into carbon dots. Bottom–up fabrication techniques, in which minor fragments polymerise and carbonise to produce carbon dots. CDs are gaining popularity due to their adaptability and simplicity of usage. Recent studies synthesise CDs using renewable, purified substances such as citric acid (Chahal, Yousefi, and Tufenkji 2020.) or plants (Jiang et al. 2019). The capacity to alter and fine‐tune the characteristics of CDs produced using renewable resources and techniques allows for the growth of various applications in fields such as bioimaging (Wan et al. 2019; Sharma, Tiwari, and Mobin 2017), fluorescent patterning inks (Bhatt et al. 2018; Sharma et al. 2018), energy applications (Gao et al. 2017), electronics (Sharma 2021), photocatalysis (Qin et al. 2013), sensing (Kumar et al. 2017) and anti‐microbial (Shahshanipour, Rezaei, and Ensa 2019).

Notably, the production of CDs with intrinsic bioactivity offers numerous methods for the development of next‐generation medications for the direct control or management of specific illnesses, owing to the preceding remarkable benefits. Numerous bioactivities, including anti‐microbial for the treatment of bacterial keratitis (Yan et al. 2017), peroxidase‐like (Shi et al. 2011), anti‐viral, anti‐cancer, and anti‐inflammatory (Hu et al. 2016) have been identified. All such benefits have prompted scientists to investigate the supplemental biomedical and pharmaceutical implications of compact discs. In particular, the mitigating effects of carbon dots, sourced from carbonised *Schizonepetae Spica*, on haemorrhages induced by *Deinagkistrodon acutus* venom, which had already given another standpoint on the exploration of the favourable properties of CDs on acute kidney injury (Sun et al. 2018).

Pain is a pervasive symptom with numerous causes (De Leon‐Casasola 2013). Currently, the management of modest to too much pain often includes the practice of opioid drugs, encompassing morphine and its synthetic derivatives. In addition, NSAID drugs such as aspirin, along with various local anaesthetics, are also employed in pain treatment protocols (Wang and Wang 2003). Nevertheless, the deleterious effects associated with these medicines are significant (Michael et al. 2011; Pasternak 2007; Al‐Swayeh et al. 2010), necessitating the development of pain‐relieving medications with fewer adverse effects. Hypotoxic CDs have the promise to be an innovative analgesic medication.

In this study, we explored the eco‐friendly synthesis of photoluminescent carbon dots employing a direct one‐step pyrolysis process, utilising natural *Cortex Phellodendri Chinensis* as the precursor material, and studied their analgesic effect in mice model by performing tail immersion, hot plate and acetic acid writhing tests.

## 2 Experimental Works

### 2.1 Chemicals

*Cortex Phellodendron Chinensis* was obtained from Beijing Qiancao Herbal Pieces Co. Ltd. (Beijing, China). Acetic acid, fetal bovine serum (FBS), trypsin, dialysis membranes (1000 Da), MTT (3‐(4,5‐dimethyl‐2‐thiazolyl)‐2,5‐diphenyl‐2H‐tetrazolium bromide), and other chemicals and solvents were procured from Sigma‐Aldrich, Shanghai.

### 2.2 Preparation of *Cortex Phellodendron Chinensis*—Carbon Dots (CCDs)

The *Cortex Phellodendron Chinensis* materials were kept in distinct ceramic crucibles and allowed in a preheated furnace at 350°C for1 h. After chilling to 25°C, the collected black remnants were pulverised into a smooth powder and simmered twice in a water bath at 90°C for a period of 1 h. The resulting mixture was then obtained by passing the mixture through a cellulose acetate (0.22 µm) membrane. Later, the contaminants were eliminated via dialysis for about 72 h. The CCD mixture was made highly concentrated and kept at 4°C until use.

### 2.3 Characterisation

A JEOL JEM‐2100 electron instrument at 200 kV was performed to examine transmission electron microscopy (TEM) results to know the morphology of the CCDs. Using CECIL, Cambridge, UV‐visible and F‐4500 fluorescence spectroscopy were used to know the ultraviolet–visible (UV‐Vis) spectrum and fluorescence of prepared CCDs, accordingly. Fourier‐transform infrared (FTIR) spectroscopy was performed by preparing a pellet with KBr and sample, which was later used for measurements using a Thermo Fisher Scientific, CA, USA instrument. We utilised a Carlo Erba EA 1108 analyser to perform elemental analyses (CHNS) on CCDs. The combustion process was conducted at 1013°C, utilising an oxygen flow rate of 15 mL/min. The size histogram and zeta potential measurements of prepared CCDs were determined with the help of Horiba Scientific Nanoparticci (SZ‐100) equipment by dynamic light scattering (DLS) analysis.

### 2.4 In Vitro Cytotoxicity

L929 cells were procured from Shanghai Cell Bank, Shanghai. After harvesting and adjusting the L929 cell concentration to 1 × 10^4^ cells/mL, the cells about 180 µL/well were inoculated in 96‐well plates and treated with 20 µL of different concentrations (200, 100, 50, 25, 12.5, 3.18 and 0.78 µg/mL) of CCDs. In a humidified incubator at 5% CO_2_ and 37°C, all cultures were allowed to incubate for 72 h. The MTT experiment was performed to determine the quantity of viable cells.

### 2.5 Intracellular Uptake

The osteosarcoma cell line MG63 was procured from Shanghai Cell Bank, Shanghai. MG63 cells were maintained in DMEM medium added with FBS (10%) and trypsin, followed by seeding at a density of 3 × 10^4^ cells/well in tissue culture plates. Following incubation for 12 h overnight in a humidified 5% CO_2_ incubator, CCD exposure was carried out (200 µg/mL) in 300 µL of media and allowed for 12 h incubation. Later, the cells were rinsed thrice with freshly prepared media. With the help of a confocal microscope, live‐cell imaging was performed with laser excitation at 488 nm in the green region.

### 2.6 Analgesic Activity of CCDs

#### 2.6.1 Animals and Their Grouping

The animals used for this investigation were male Kunming mice, weighing about 28 ± 1.0 g. The mice had unrestricted water and food available to them while being maintained at a steady 25°C room temperature as well as a 12 h light/dark cycle.

All mice were randomly divided into five groups, with seven mice in each group, as follows:

Group 1: Control group (0.5 mL of normal saline)
Group 2: Mice administered with 5 mg/kg of morphine (positive control)
Group 3: Mice administered with 1.25 mg/kg of immunoglobulin (ig) for chemical nociception and CCDs for thermal and hot plate tests (low)
Group 4: Mice administered with 2.5 mg/kg of ig for chemical nociception and CCDs for thermal nociception and hot plate tests (medium)
Group 5: Mice administered with 5 mg/kg of ig for chemical nociception and CCDs for thermal nociception and hot plate tests (high)

After 1 h of administering drugs, acute versatile pain was obtained by intraperitoneal injection of normal saline (0.7% v/v), acetic acid (0.1 mL/10 g).

#### 2.6.2 Chemical and Thermal Nociception Study

By following the acetic acid writhing experiment, pain responses in reaction to chemical stimulus were evaluated. After receiving acetic acid injections for five minutes, the animals were split up separately to notice the writhing behavioural responses. The evaluation period was about 15 min long. The anti‐nociceptive action of CCDs was quantified as writhing counts (Rudrapal et al. 2022).

#### 2.6.3 Hot Plate Test

The mice were arbitrarily allotted to five groups. To minimise the likelihood of transfer effects and to mitigate possible influences from olfactory or visual stimuli, the mice in the hot plate test were acclimatised to the quasi‐device for 5 min before they performed the experimental test. The temperature was then maintained at 50°C, and the animal was positioned on the hot plate to assess their ability to respond both after and before the administration of the drug. When nociception first manifested, which resulted in a lick or bite at hind paws, response latency(s) were observed. Mice with baseline responses longer than 30 s were excluded from the test, and a maximum lower limit of 50 s was set to avoid hind paw injury following administering drugs. After each evaluation in the test, the hot plate was thoroughly cleaned using 75% ethyl alcohol.

#### 2.6.4 Mouse Tail Immersion Test

The tail immersion test was additionally employed to evaluate the CCDs anti‐nociceptive effect. The animals were physically constrained using a porous plastic tube with their tails facing the controller. Also, every mouse's distal tail (about 3 cm) was placed in a water bath that was kept at 55°C to create a harsh heat stimulus. Relative to this, the response time was measured as the withdrawal delay in reaction to the heat stimulus, as indicated by a forceful removal of the tail from the heated water. Mice with baseline reaction times greater than 5 s were disqualified from the experiment. Furthermore, the highest cut‐off time was maintained at 10 s to protect the mouse's tail.

### 2.7 Statistical Significance

IBM SPSS Statistics was used to conduct statistical analyses (version 20). Data characterised by uniform variances and a normal distribution were presented as means ± standard deviation. For conducting multiple comparisons, an ANOVA was employed, followed by the test for the least noteworthy difference. *p* values not more than 0.01 were considered for statistical analysis.

## 3 Results and Discussion

### 3.1 Characterisation of CCDs

**FIGURE 1:**
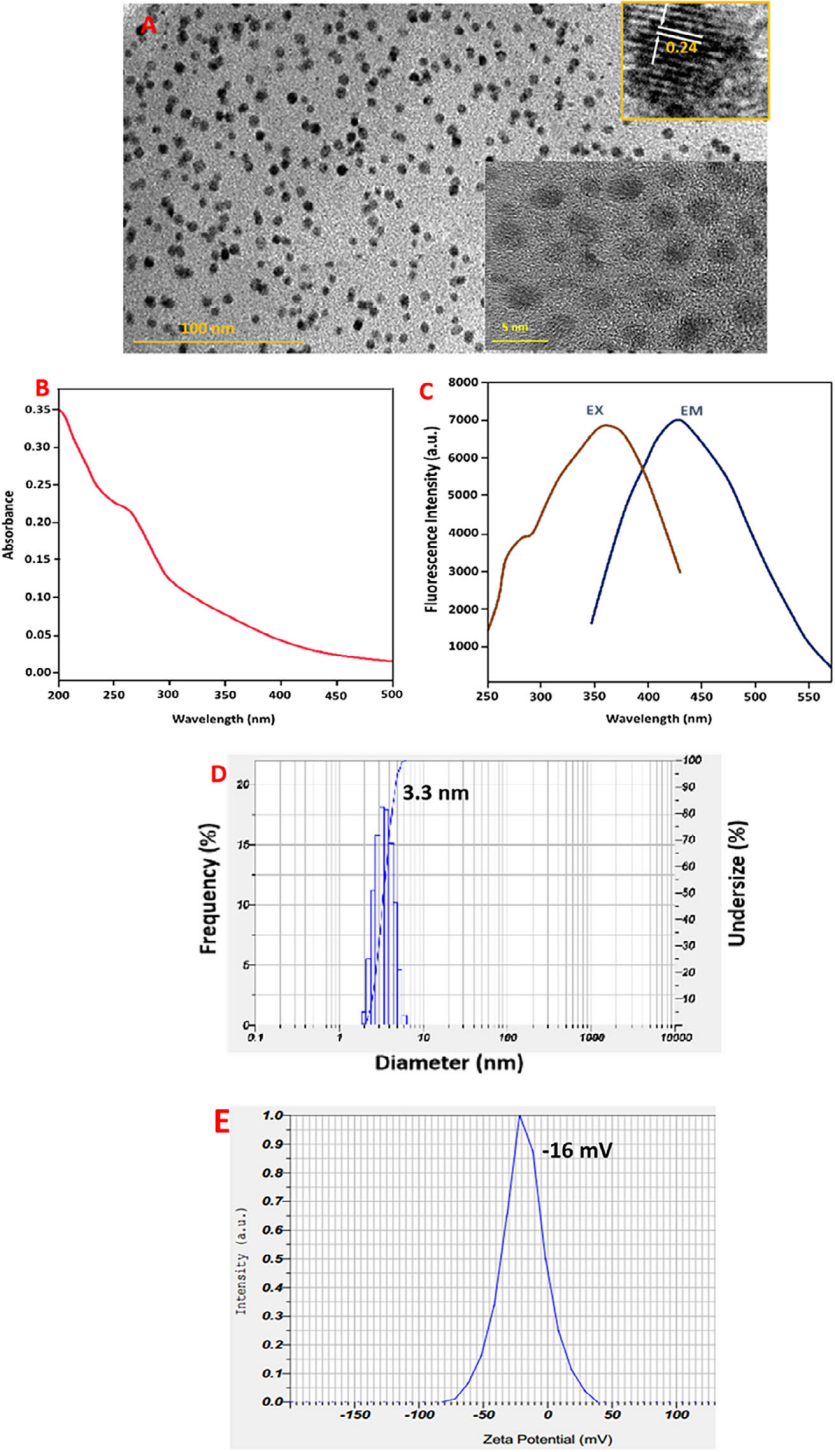
(A) HR‐TEM images, (B) UV–visible spectra, (C) fluorescence spectra, (D) DLS size distribution and (E) zeta potential of prepared CCDs showing the formation of ultra small, stable carbon dots.

The shape and size distribution of CCDs have been confirmed by HR‐TEM (Figure 1A). The produced CCDs were homogenous in size, with spherical in shape and the majority measuring about 3.3 nm without aggregates. As depicted in Figure 1A and insets, the results of HRTEM revealed the lattice fringes of about 0.24 nm of the synthesised CCDs that correlated to graphitic carbon structure. As illustrated in Figure 1C, the CCDs displayed a unique blue fluorescence with a most significant band found at 445 nm and subsequent excitation at 370 nm. Consequently, the UV‐Vis results of the CCDs revealed a pronounced absorption band at 265 nm, owing to the π─π* transition of attached organic substances on the surface of CCDs (Figure 1B). On the other hand, Figure 1D represents the DLS size histogram, showing an average size of about 3.3 nm, which agrees with the HR‐TEM results obtained. Furthermore, the stability of prepared CDDs was found to be −16 mV, which directs the existence of negatively charged ─COOH and ─OH functionalities on the surface of the prepared CDDs.

**FIGURE 2:**
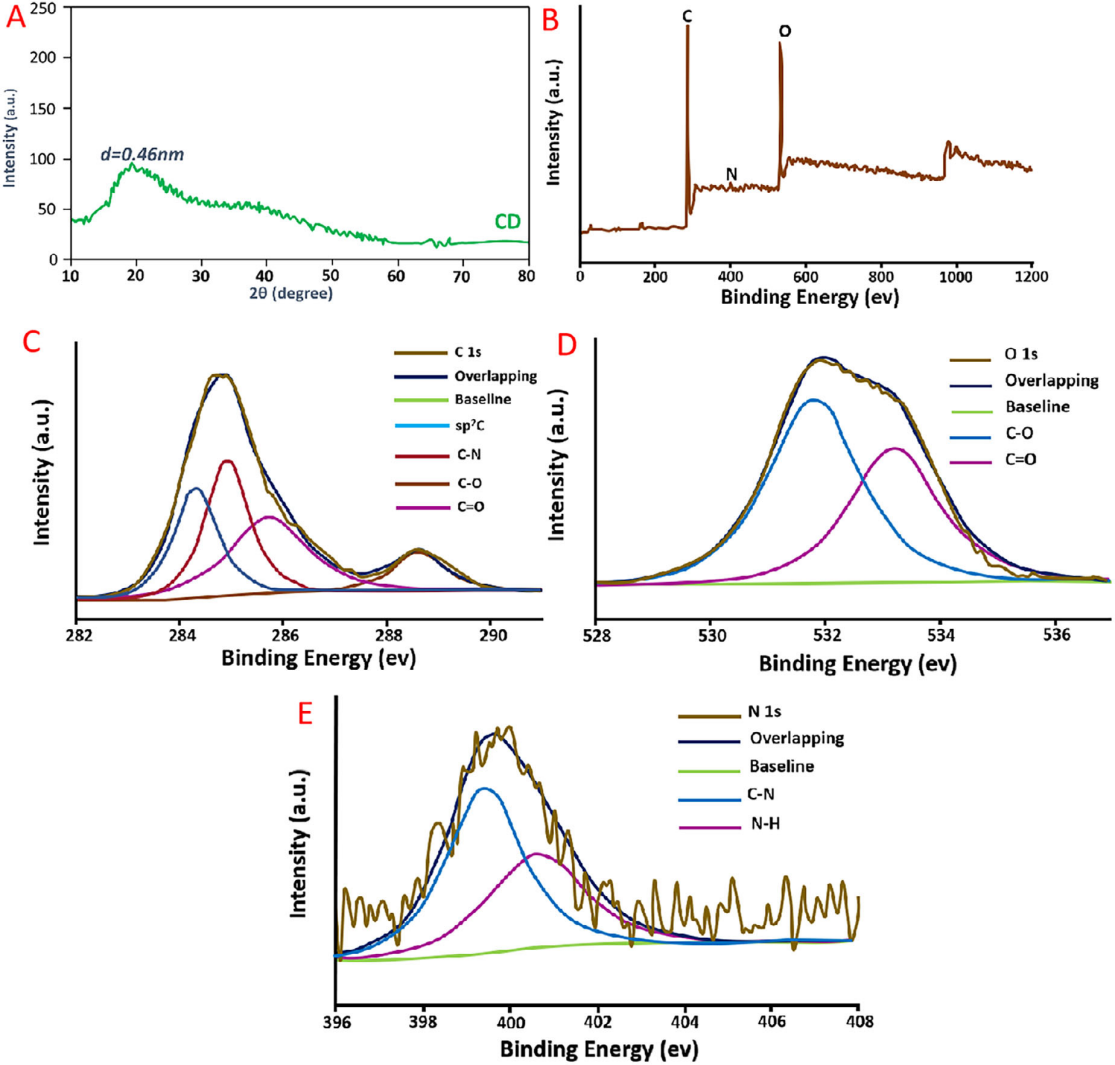
(A) XRD pattern, (B) XPS broad spectrum, (C) C1s spectrum and (D) O1s spectrum of CCDs showing the formation of water‐soluble CCDs.

**FIGURE 3:**
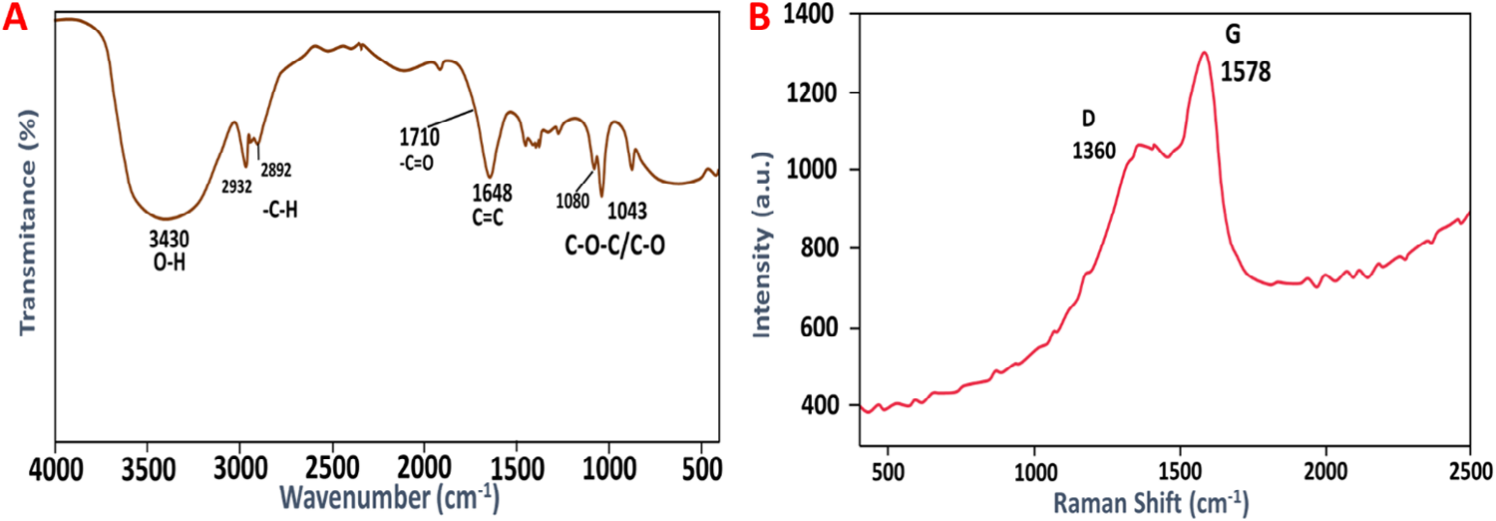
(A) FTIR spectrum and (B) Raman spectrum of showing the surface functionalization of formed CCDs.

Typical XRD patterns of prepared CCDs are depicted in Figure 2A. It is shown that the peak is centred at 22, which corresponds to the (211) index. The interlayer spacing corresponding to CCDs is found to be 0.46 nm, which indicates their amorphous nature. In addition, the structure of CCDs has been studied using XPS analysis (Figure 2B–E). Figure 2B displayed a spectrum with three distinct peaks observed at energy levels of 284.7, 399.6 and 532.2 eV. The observed peaks are indicative of the distinct binding energies associated with C1s, N1s and O1s. The C1s spectra with great resolution (Figure 2C) showed the presence of carbons in five different chemical habitats. These environments correspond to C═O (288 eV), sp2 (C═C) at 284.1 eV, sp3 (C─C and C─H) at 285.5 eV, ─COOH at 283.0 eV, and C─OH/C─O─C at 285.7 eV. These results suggested the composition of the carbon dots (CCDs) includes functional groups like carboxylic acid, hydroxyl, epoxy and carbonyl. In contrast, the O1s peak (Figure 2D) is predominantly comprised of two subpeaks positioned at 531.8 eV (representing C─O bonds) and 533.2 eV (associated with C═O bonds). The N1s spectra (Figure 2E) displayed two clearly distinguishable peaks at 399.4 and 400.6 eV, signifying the existence of C─N and N─H bonds, respectively. Conversely, the elemental analysis results indicated that the synthesised CCDs consist of nitrogen (9.80%), hydrogen (4.58%), carbon (56.67%), sulphur (0.81%) and oxygen (calculated, 28.14%). However, these results were supported by FTIR analysis, where the existence of peaks is found at 2892 cm^−1^ (C─H), 1710 cm^−1^ (C═O), 1648 cm^−1^ (C═C), 1080 cm^−1^ (C─O─C) and 3430 cm^−1^ (O─H) groups, which contributes to the enhanced solubility of the CCDs in water (Figure 3A). In addition, Raman spectroscopy results are presented in Figure 3B. The detection of the D‐band at 1360 cm^−1^ can be ascribed to the vibrations of carbon atoms with unpaired bonds, while the G‐band, observed at 1578 cm^−1^, arises from the vibrations of sp2 hybridised carbon in a two‐dimensional hexagonal lattice of graphite clusters (refer to Figure 3B). Furthermore, the ID/IG ratio is equal to 0.75, which suggests the amorphous state of the CCDs.

### 3.2 In Vitro Cytotoxicity

**FIGURE 4:**
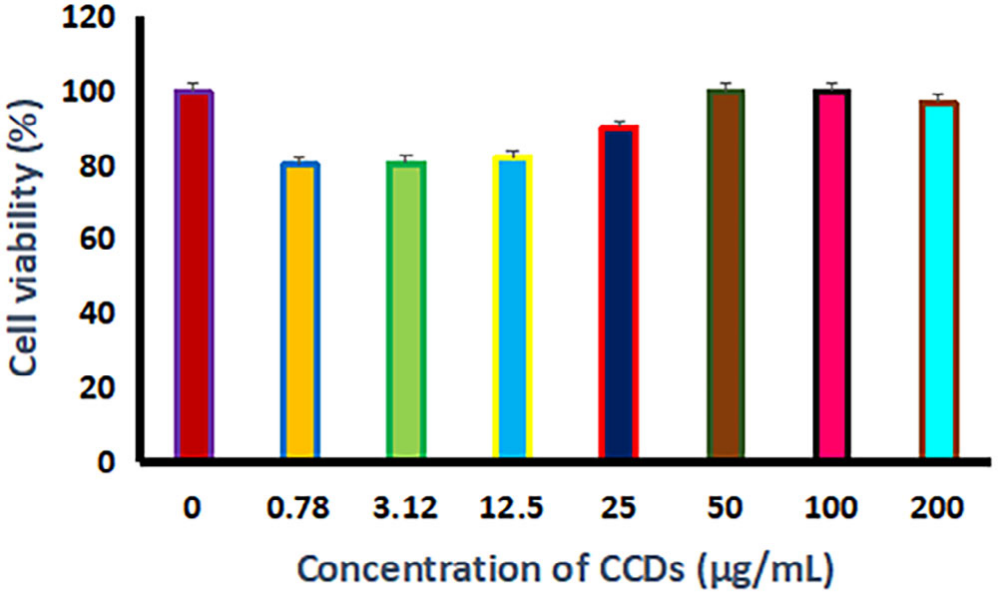
Cell viability of L929 cells treated with various concentrations of CCDs showing their biocompatibility. *p* ≤ 0.01 was considered as statistically significant.

From the cytotoxicity results, it is found that no substantial toxicity to L929 cells was found and that they are less toxic even at higher CCD concentration (200 µg/mL) at a longer incubation time of about 72 h, suggesting that the prepared CCDs are suitable for in vitro and in vivo use (Figure 4) (Li et al. 2020).

However, the above‐mentioned results were validated using the live/dead experiment. In the Live/Dead assay, cells cultured on an eight‐well IBIDI µ‐slide were treated with Calcein‐AM (3 µg/mL) and propidium iodide (6 µg/mL). This staining procedure was carried out for 30 min at an approximate temperature of 37°C. After the incubation period, DPBS was used to wash the cells and promptly analysed using LSM fluorescence microscopy. The presence of green staining signifies the intactness of the cell membrane and, consequently, the existence of viable cells. Conversely, the presence of red staining indicates a disturbance in the membrane and the staining of the nucleus by the propidium iodide.

**FIGURE 5:**
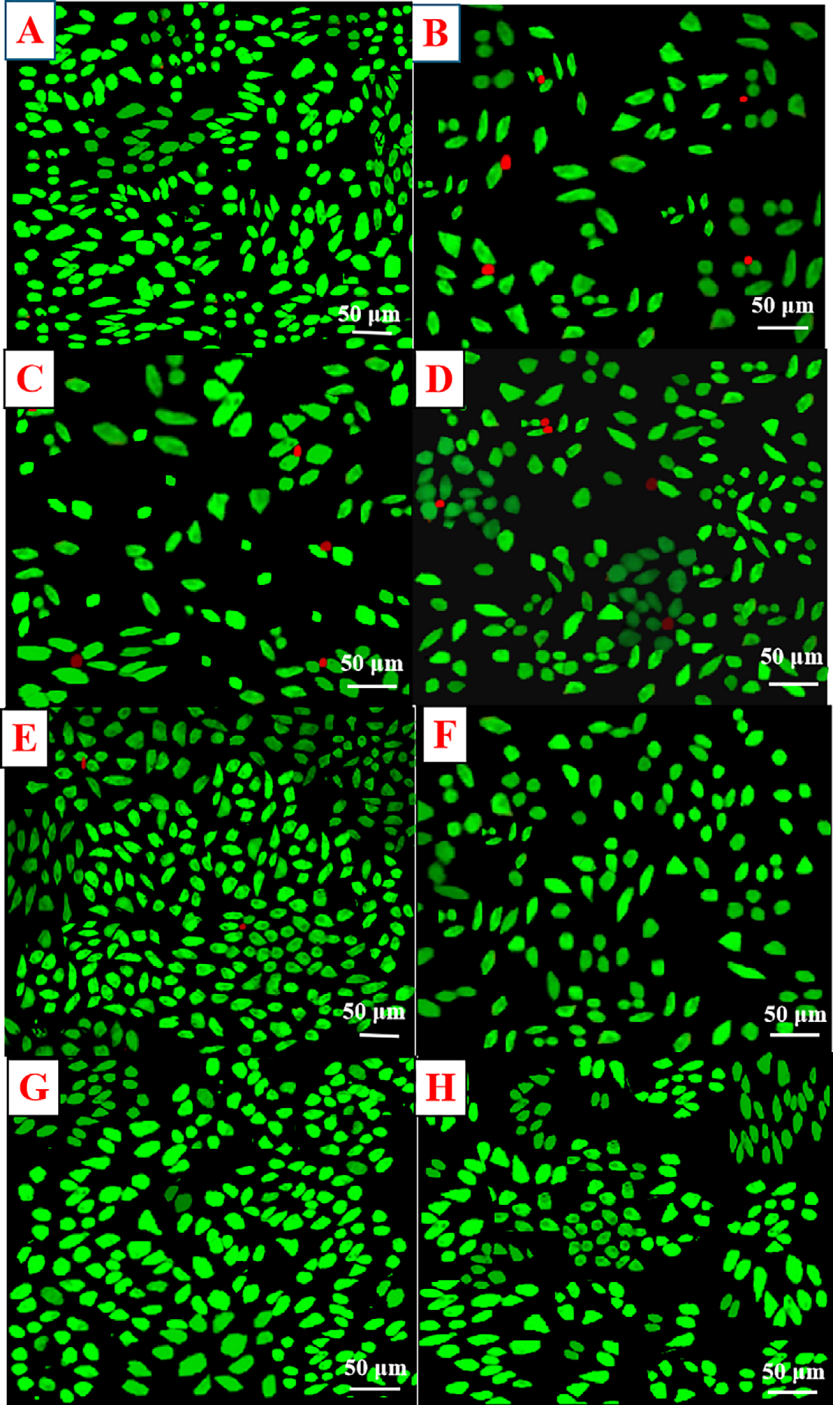
Membrane integrity of L929 cells after incubation with different concentrations of CCDs (A) Control, (B) 0.78 µg/mL, (C) 3.12 µg/mL, (D) 12.5 µg/mL, (E) 25 µg/mL, (F) 50 µg/mL, (G) 100 µg/mL and (H) 200 µg/mL of CCDs.

Our current research found that the synthesised CCDs maintained the membrane integrity of L929 cells at certain concentrations, evident from the green staining observed. Nevertheless, at CCD concentrations of 0.78 and 3.12 µg/mL, there was a noticeable disruption in the L929 cell membranes, as demonstrated by the red staining (Figure 5). The increase in cell viability with an increase in CCD concentration is because of the proliferation that arises at this concentration, apparently due to the hydrophilic functionalities and high oxygen content of the prepared CCDs, which endorse cell adhesion in the hydrophilic environment (Oh et al. 2014).

The lower cytotoxic effects and good biocompatibility levels of CDs are mainly because of their organic nature and precise physicochemical features. For instance, CDs derived from various natural sources, such as glucose, chitosan, carbohydrates, citric acid and amino acids, consistently establish greater biocompatibility with low cytotoxicity (Sri et al. 2018; Iravani and Varma 2020; Raveendran and Kizhakayil 2021; Dias et al. 2019; Ross et al. 2020). Furthermore, the smaller size of CDs (< 10 nm) possibly improves their absorption by cells. The surface of CDs can also be modified to either decrease toxicity or augment biocompatibility (Zhang et al. 2021; Yan et al. 2018; Díez‐Pascual 2022). For example, coating CDs with polyethylene glycol leads to lower cytotoxicity in comparison to uncoated CDs (Suk et al. 2016; Yang et al. 2009; Li et al. 2020). Similarly, the synthesis CDs using *Cortex Phellodendron Chinensis* may lead to surface coating, which might neutralise surface charge that is more compatible with cellular surfaces. However, the exact mechanism of cytotoxicity of CDs is unclear to date.

### 3.3 Cellular Uptake Studies

**FIGURE 6:**
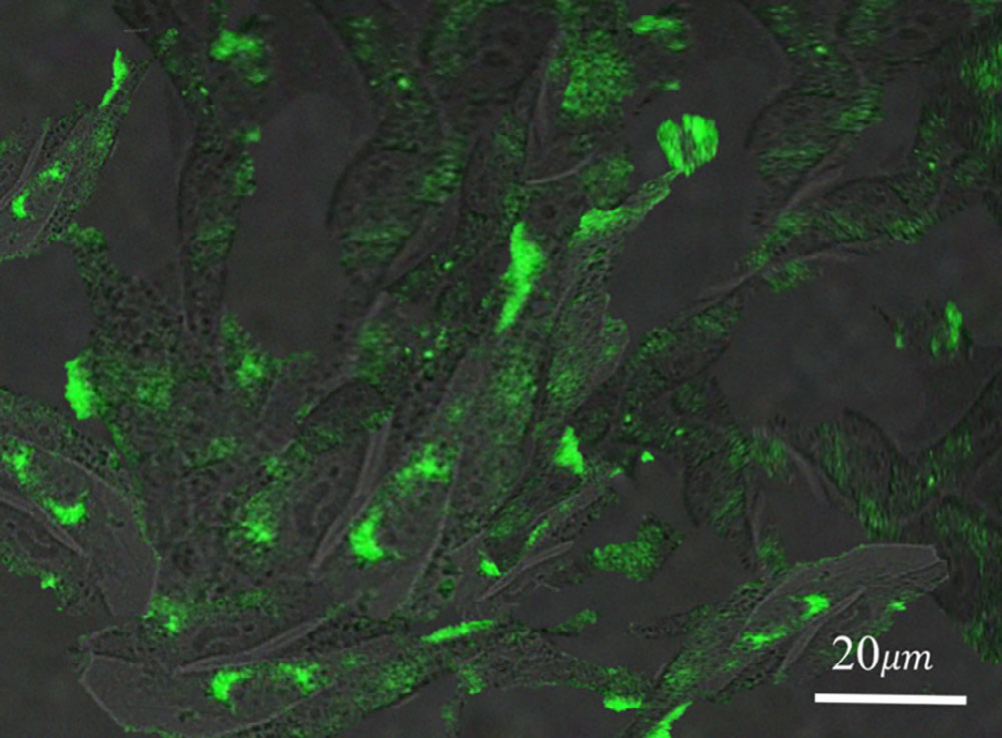
Confocal microscopic images of MG‐63 cells showing the aggregation of green, fluorescent CCDs within the cells.

Figure 6 depicts confocal microscopic images of MG‐63 cells exposed to CCDs at 488 nm excitation wavelength. The phase contrast images of MG‐63 cells at wavelength 488 nm clearly depict the aggregation of green, fluorescent CCDs within the cells while leaving nuclei unobscured (Figure 6). Furthermore, the luminescence intensity is unaffected by continuous excitation. All these results suggested that the prepared CCDs are a suitable alternative for semiconductor QDs for bioimaging applications.

### 3.4 Anti‐Nociceptive Effect of CDs

#### 3.4.1 Chemical Pain and Thermal Pain

**FIGURE 7:**
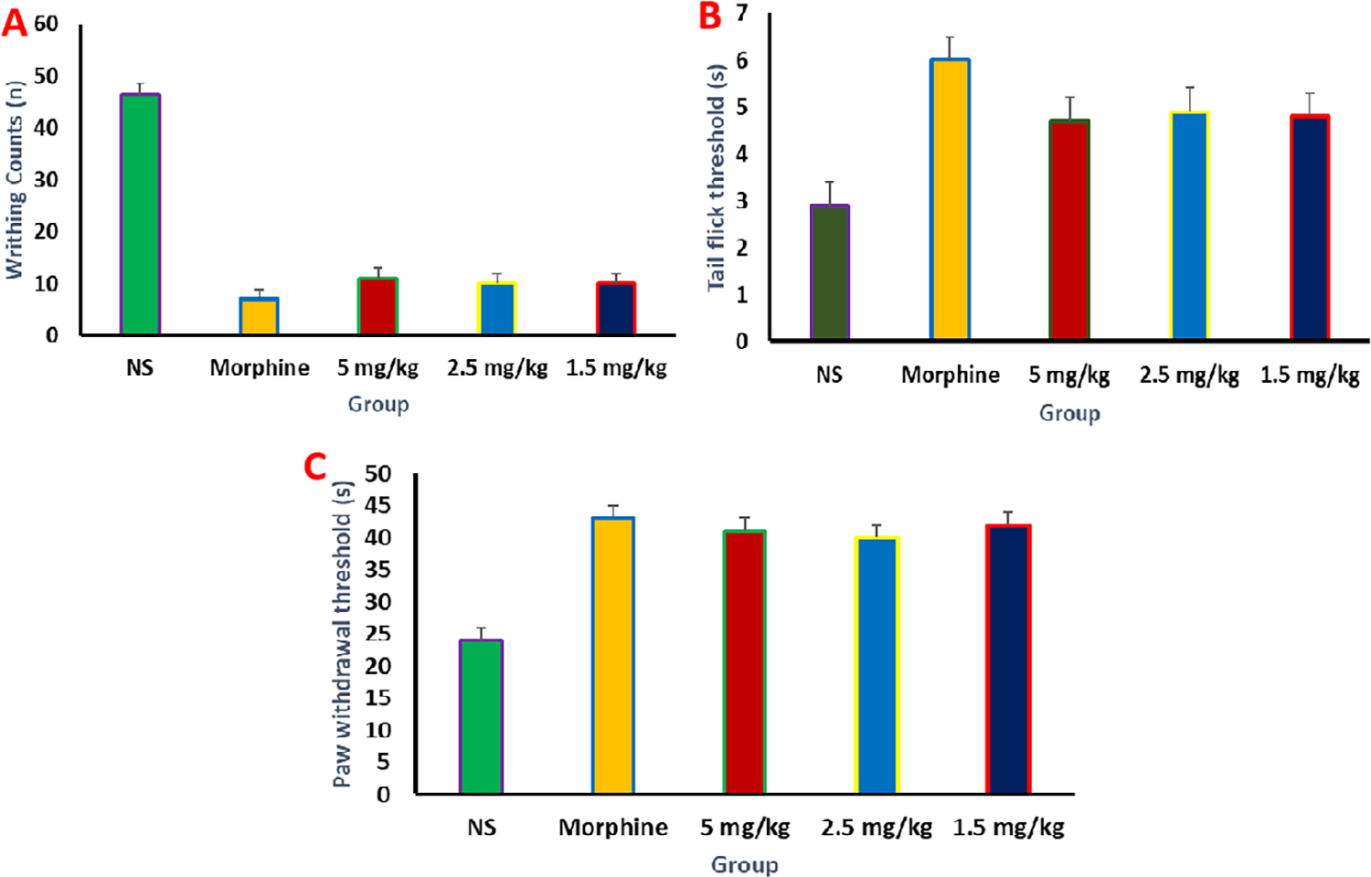
The anti‐nociception activity of CCDs at different concentrations 1.25, 2.5 and 5 mg/kg. (A) Acetic acid writhing, (B) hot‐plate and (C) tail‐immersion tests. *p* ≤ 0.01 was considered as statistically significant.

Figure 7A illustrates the quantification of writhing responses employed to evaluate the anti‐nociceptive effects of the carbon dots (CCDs) in experiments involving acetic acid‐induced writhing. Substantial analgesic effects were noted in the low (10 ± 3), medium (10 ± 3) and high (12 ± 3)b concentrations of CCDs, along with the morphine group (6 ± 3 counts), when compared to the normal saline treatment (45 ± 13). Furthermore, Figure 7B,C displayed the outcomes related to the hindpaw and tail‐flick withdrawal reflexes, respectively. These figures are indicative of the anti‐nociceptive efficacy of CCDs as observed in the hot plate and tail immersion tests. Specifically, mice exposed to CCDs at high (hot plate: 40 ± 7 s; tail immersion: 4 ± 1 s), medium (hot plate: 40 ±7 s; tail immersion: 5 ± 1 s) and low (hot plate: 42 ± 5 s; tail immersion: 5 ± 1 s) were found. All these results indicated a significantly improved response latency towards the thermal stimulus in both models, which suggests the enhanced anti‐nociceptive activity of CCDs. Notably, there was no substantial distinction between positive control and CCD treatments in either tail‐flick response latencies or hind paw withdrawal (hot plate: 43 ± 4 s; tail immersion: 6 ± 1 s).

**FIGURE 8:**
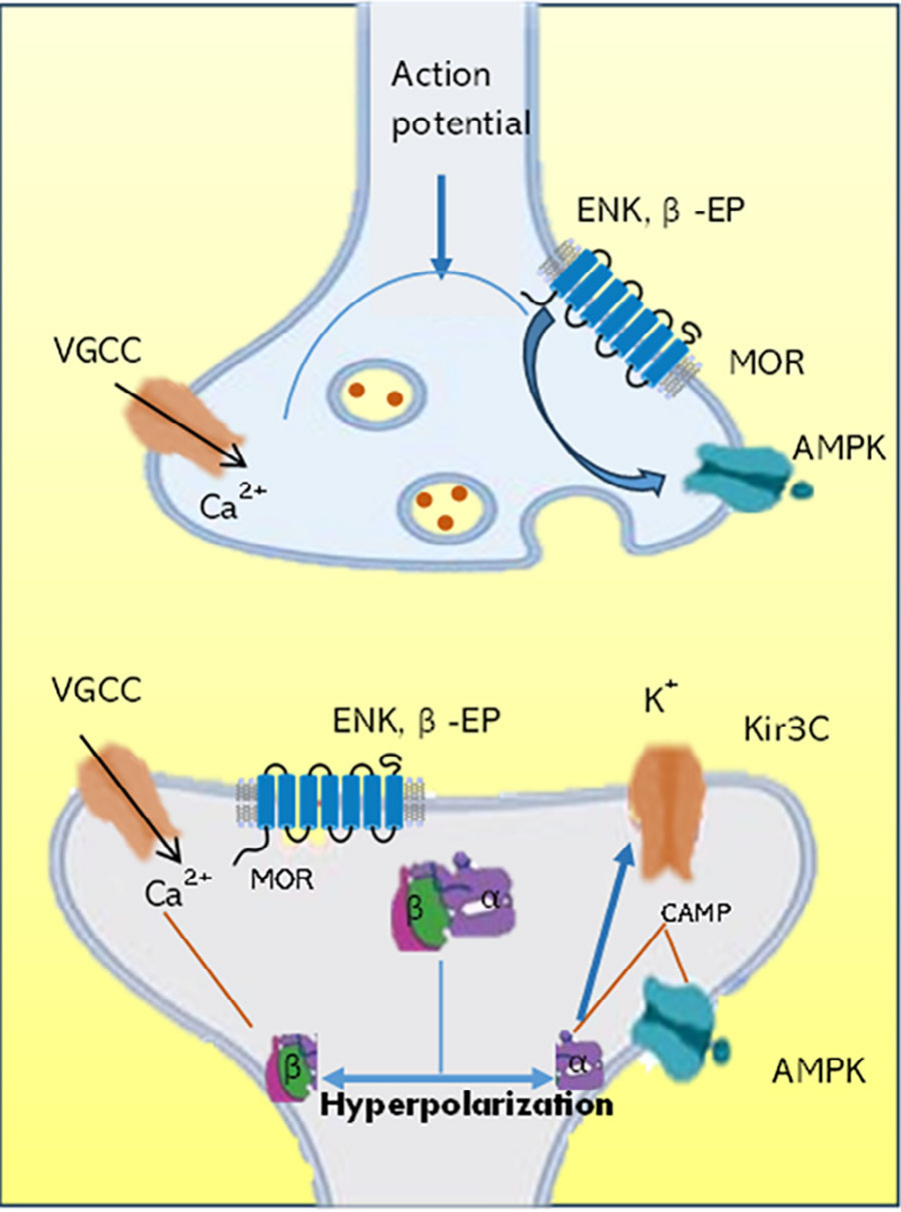
The mechanism of analgesic action of opioids binding to MOR at neural terminal.

The CCDs exhibited a substantial anti‐nociceptive effect in all of the dosages that were evaluated in the study. Due to the substantial decrease in nociceptive thresholds observed in both the tail immersion and hot plate experiments, it is possible that CCDs may affect both spinal and supraspinal pain pathways. The opioidergic mechanism is crucial to pain management (Giordano 2005). The investigation of analgesic mechanisms was introduced by the exploration of the endogenous opioid peptide ENK in 1975 and ß‐EP in 1976 (Han 2004; Misra et al. 2017). ENK is prevalent in the spinal cord's primary afferent terminals, and it may be triggered by painful peripheral stimuli (Ruda, Bennett, and Dubner 1986). Numerous investigations have shown a correlation between elevated ENK levels and pain management (Al‐Amin et al. 2003). Whenever the result is extreme vigorous exercise and agitation, ß‐EP is discharged into the pituitary gland and hypothalamus (Lee et al. 2009), comparable to the reaction of ENK in the regulation of pain. The possible mechanism for the anti‐nociceptive effect of CCDs may be because of the elevated levels of ENK and ß‐EP during thermal stimulation‐induced analgesia during both hot plate and tail immersion tests (Figure 8). Nonetheless, future studies are needed to know the comprehensive molecular mechanisms of the analgesic activity of CCDs. However, similar nociception studies were performed using Capsaicin transfersomes, which bind to nociceptors in the skin, causing an initial excitation of the neurones and a period of enhanced sensitivity to noxious stimuli, usually perceived as itching, pricking or burning sensations (Kumar, Rudrapal, and Mazumder 2014; Sarwa et al. 2014).

## 4 Conclusions

In conclusion, in the current investigation, green photoluminescent CDs were produced through a one‐step direct pyrolysis method using natural *Cortex Phellodendron Chinensis* material. TEM images showed that the produced CCDs were homogenous in size, spherical in shape and the majority measuring about 3.3 nm without aggregates. The present study exhibited proficient anti‐nociceptive action of the combination of CCDs with the extract of *Cortex Phellodendron*. Furthermore, these results demonstrated the beneficial effects of CCDs from the *Cortex Phellodendron* through anti‐nociceptive activities. L929 cells exhibited higher cell viability when treated with prepared CCDs. In addition, to the best of our understanding, the results presented in this paper showed that CCDs exhibited an important analgesic effect, which may be due to stimulation of the opioidergic system. Consequently, CCDs appear to be a viable analgesic alternative for traditional analgesic candidates in pain management.

## Author Contributions

**Huimin Peng**: validation, data curation, investigation. **Xingxing Zeng**: formal analysis, visualization. **Songbai Li**: writing–original draft, writing–review and editing, conceptualization, methodology, project administration. Xin Wang: formal analysis, supervision, software.

## Ethics Statement

The research carried out in this article with animals was in line with the principles and standards outlined in the institutional animal ethical committee of Xiangyang Central Hospital, Xiangyang City, Hubei Province. All the animal experiments were conducted according to their guidelines and regulations.

## Conflicts of Interest

The authors declare no conflicts of interest.

## Data Availability

The data pertaining to the conclusions of this study can be obtained from the respective authors upon a reasonable request.
